# Study on Asymmetric Vibrational Coherent Magnetic Transitions and Origin of Fluorescence in Symmetric Structures

**DOI:** 10.3390/molecules28186645

**Published:** 2023-09-15

**Authors:** Lulu Sun, Ning Li, Ji Ma, Jingang Wang

**Affiliations:** Liaoning Provincial Key Laboratory of Novel Micro-Nano Functional Materials, College of Science, Liaoning Petrochemical University, Fushun 113001, China; llsun1002@163.com (L.S.); li18340352037@163.com (N.L.)

**Keywords:** chiral molecule, Raman spectrum, ECD, CDD, TEDM/TMDM, ROA

## Abstract

In this work, the physical mechanisms of three highly efficient circularly polarized luminescent materials are introduced. The UV–vis spectra are plotted; the transition properties of their electrons at the excited states are investigated using a combination of the transition density matrix (TDM) and the charge difference density (CDD); combining the distribution of electron clouds, the essence of charge transfer excitation in three structures is explained. The resonance Raman spectrum of the three structures at the S_1_ and S_2_ excited states are calculated. The M, M-4 and M, M-5 structures are found to produce novel chirality by electronic circular dichroism (ECD) spectrum, and the reasons for the chirality of the M, M-4 and M, M-5 structures are discussed by analyzing the density of transition electric/magnetic dipole moments (TEDM/TMDMs) in different orientations. Finally, the Raman optical activity (ROA) of M, M-4, and M, M-5 are calculated, and the spectra are plotted. This study will provide guidance for the application of carbon-based nanomaterials in organic electronic devices, solar cells, and optoelectronics.

## 1. Introduction

Carbon nanomolecules have a wide range of applications in photoelectric sensors [1,2,3,4,5], materials science [6], and novel energy sources [7] due to their excellent physicochemical properties. Helical polymers are macromolecules widely found in nature, such as DNA and protein biomolecules, and have helical conformation. Inspired by the helical structure of nature, the study of artificial helical polymers has attracted a great deal of research interest. Chiral materials constructed from chiral helical polymers are particularly interesting [8], and they have important application prospects in fields such as chiral self-assembly [9,10], asymmetric catalysis [11,12], chiral transfer [13,14], chiral recognition [15,16], chiral optoelectronic materials [17], circular polarization luminescence (CPL) [18], and fluorescent materials [19,20].

Perylene diimides (PDIs) are common π-conjugated molecules, and large conjugation planes facilitate the formation of supramolecular assemblies by π-π stacking and can adjust the properties of supramolecular assemblies by substituents at the imide, bay, or ortho positions [21]. In 1913, Friedlander synthesized the first perylene-based derivatives [22], and since, more and more chiral PDIs helical polymer molecules have been synthesized and studied. PDIs are widely used in the fabrications of organic optoelectronic devices due to their high structural stability, simple structural functionalization, and excellent light-trapping properties in the visible region [23,24,25]. The introduction of chirality allows the application of PDIs and their supramolecular assemblies for enantiomeric recognition, CPL emission, and CPL detection [26,27]. In 2022, Wang et al. constructed a PDIs dimer (tetrarylene-based double π-helix, named TH molecule) [28], an efficient CPL material, and achieved fine-tuning of the transition electric/magnetic dipole moments (TEDM/TMDMs) of this chiral helical polymer, finally obtaining a material system with both high luminescence asymmetry factor and high luminescence efficiency. The chiral carbon nanomolecule has D_2_ symmetry, and thanks to its rigid double π-helix backbone and the introduction of bay-site nitrogen heterocycles, the luminescence asymmetry factor $\left|g_{\mathrm{lum}}\right|$ is increased from 0.01 to 0.03, and the luminescence efficiency $\Phi_{\mathrm{PL}}$ is between 41 and 62%, which leads to a circularly polarized luminescence ($B_{\mathrm{CPL}}=\varepsilon_{\lambda }\times \Phi_{\mathrm{PL}}\times \left|g_{\mathrm{lum}}\right|/2$) also reaching 573.4 M^−1^ cm^−1^, which is one of the highest values reported for chiral small molecules. This chiral molecular system has excellent CPL properties expected to be applied in chiral optoelectronic materials, providing new ideas for the rational design of new efficient CPL materials.

This study delved into the theoretical analysis of the UV–vis spectrum, transition density matrix (TDM), and charge difference density (CDD) of three structures. The investigation aims to explain the charge transfer excitations of these structures by examining the distribution of the electron cloud. Then, we theoretically studied the resonance Raman spectrum, ECD, TEDM/TMDMs, and the Raman optical activities (ROAs) based on quantum chemical calculations and wave function analysis. This study will guide the application of carbon-based nanomaterials in organic electronic devices, solar cells, and optoelectronics.

## 2. Results and Discussion

**Figure 1:**
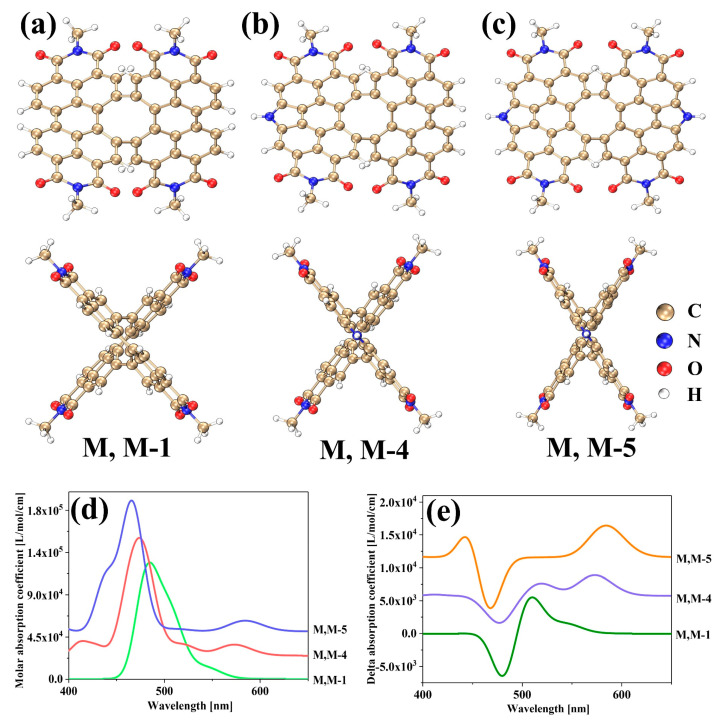
The 3D molecular structures (**a**–**c**), UV–vis spectrum (**d**) and ECD spectrogram (**e**).

Scientists have recently been paying attention to the development of new molecular carbon materials that have excellent properties in optical, electrical, and magnetic aspects, which are combined with good processing performance and stability. In 2022, Wang et al. reported a double π-helix structure that was based on a cyclooctatetraene-embedded PDI dimer with D_2_ symmetry [28]. Figure 1a–c shows the schematic structures and side views of the three molecules, while Figure 1d displays the UV–vis spectrum. Figure S1a indicates that there is no nitrogen heterocycle at the bay region in the M, M-1 structure. It also shows that the positions of S_1_ and S_2_ excited states are closer. In the M, M-4 structure, a nitrogen heterocycle is added at the bay position, creating a pyrrole. This results in a red shift in the absorption peak for the S_1_ excited state and a blue shift in the absorption peak for S_2_. The S_1_ and S_2_ excited states become more separated, causing a new absorption peak to appear and an increase in absorption intensity. In the M, M-5 structure, two nitrogen heterocycles are added at the bay, causing the formation of two pyrroles. The distribution of excited states S_1_ and S_2_ is comparable to that of the M, M-4 structures, and new absorption peaks are visible, as shown in Figure S1c. The intensity of absorption in the S_2_ excited state decreases. The emergence of a new peak in the UV–vis absorption spectrum is directly linked to the introduction of nitrogen. The introduction of nitrogen heterocycles in the bay position can cause the splitting of excited states, leading to the creation of new absorption peaks.

**Figure 2:**
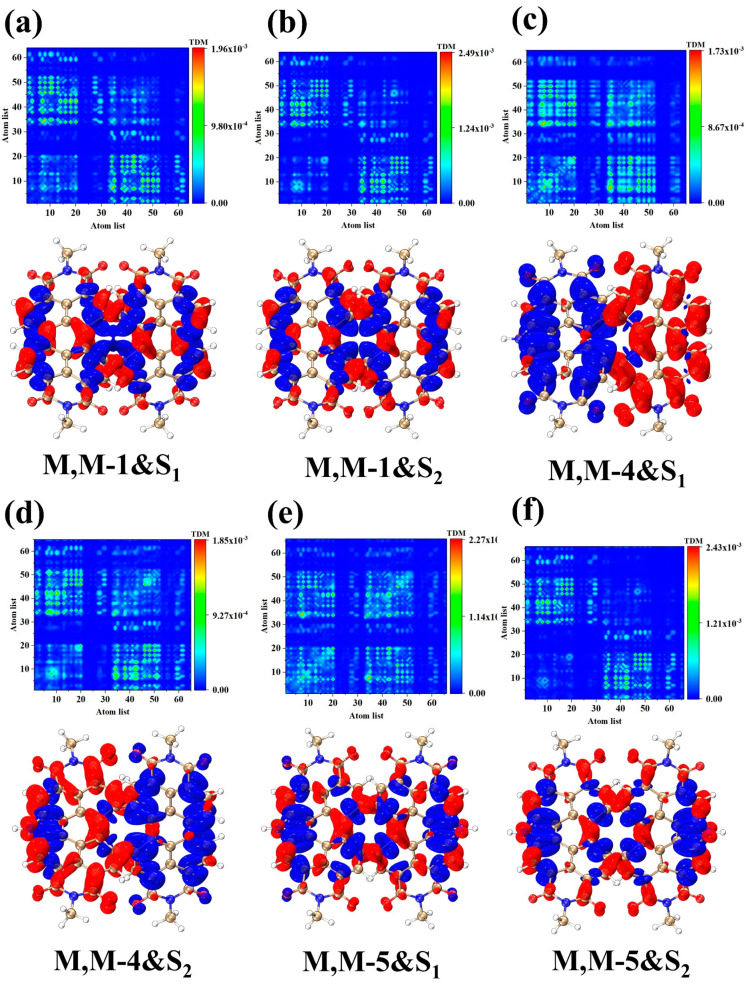
TDM and CDD diagrams of S_1_, S_2_ excited states in M, M-1 (**a**,**b**), M, M-4 (**c**,**d**) and M, M-5 (**e**,**f**). The red (blue) isosurfaces represent electron (hole) density, respectively.

Due to the difference in absorption intensity and the splitting of the absorption peaks, the charge transfer in the S_1_ and S_2_ excited states is necessarily affected by the appearance of new absorption peaks. Therefore, the study was carried out with the S_1_ and S_2_ excited states corresponding to the two split absorption peaks. Figure 2 shows the TDM and CDD plots of S_1_ and S_2_ excited states for three structures. As can be seen from the CDD plots of structure M, M-1 in S_1_ and S_2_ excited states (Figure 2a,b), the distributions of electron and hole densities are roughly left-right symmetric, which depends on the symmetry of the M, M-1 structure itself. It is apparent from the TDM diagram that charge-transfer excitations dominate both excited states, as the transition densities are mainly in the off-diagonal region. From the CDD plots of structure M, M-4 in S_1_ and S_2_ excited states (Figure 2c,d), the electrons of S_1_ are mostly distributed in the right segment of the structure and the holes are mostly distributed in the left segment of the structure, while the distribution of electron-hole density in S_2_ excited states is opposite to that of S_1_. The electron and hole densities of the two excited states are separated from left to right. This is caused by the disruption of the molecule’s symmetry after the nitrogen heterocycle is introduced at the bay position, which leads to different electronic excitation behavior. Combined with the TDM diagram, it can be seen that the transition densities of S_1_ and S_2_ excited states appear mainly in the non-diagonal region, with only a small amount of transition densities at the diagonal, which indicates that both excited states are dominated by charge transfer excitations accompanied by weak local excitations. By investigating the CDD plots of structures M, M-5 in their respective S_1_ and S_2_ excited states (Figure 2e,f), it is apparent that the distribution of electrons and holes returns to a symmetric pattern from left to right. This is a result of the introduction of two nitrogen heterocycles in the bay position, which restore symmetry to the molecule’s structure.

**Figure 3:**
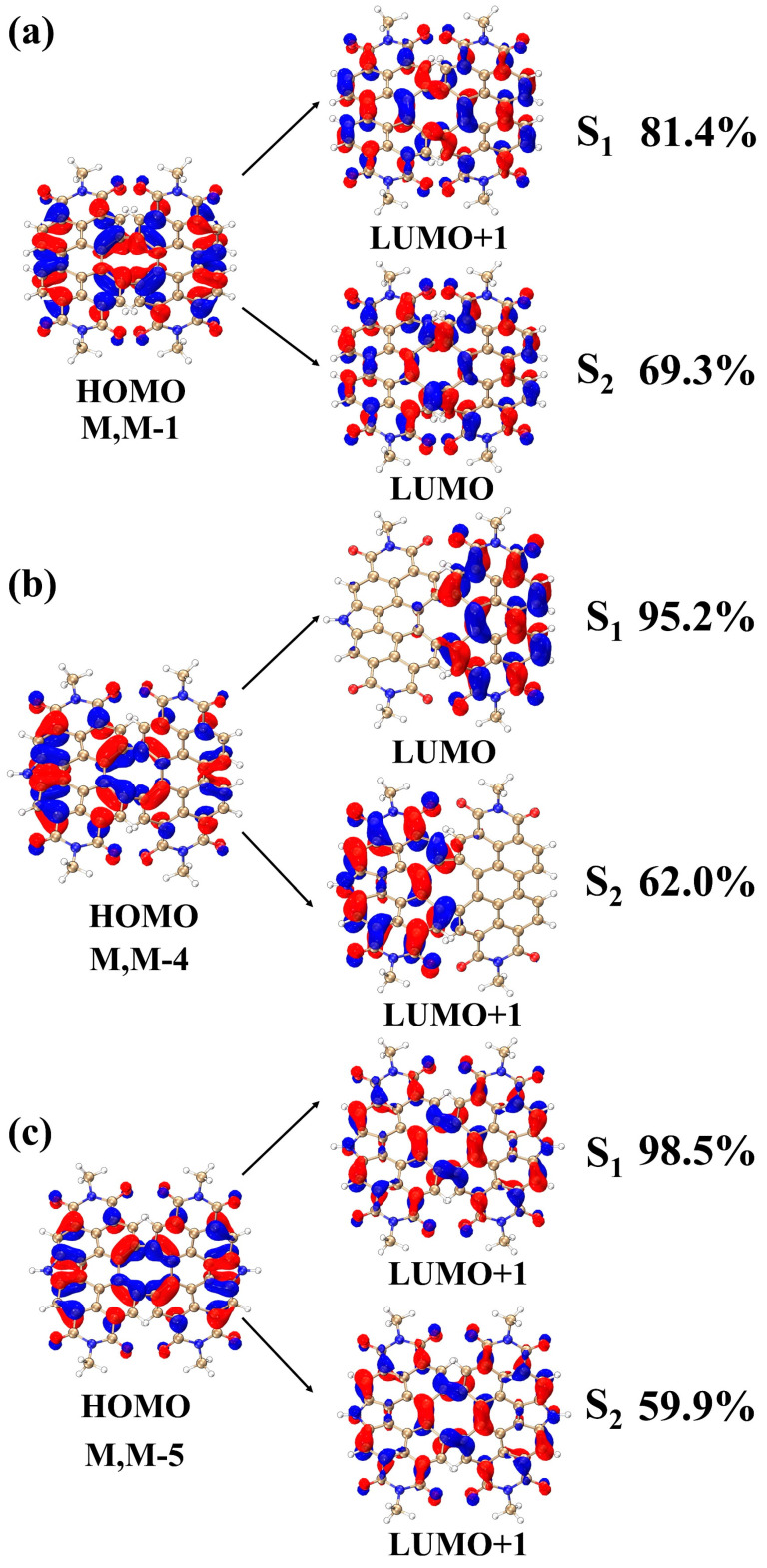
Electron cloud distribution of the main contributing orbitals of the S_1_, S_2_ excited states of the three structures (**a**–**c**). Red and blue regions denote the positive and negative orbital phases, respectively.

The excitation modes of the S_1_ and S_2_ excited states are analyzed based on their TDM and CDD diagrams. It can be seen that the appearance of a new absorption peak has an impact on the excited state charge transfer, which is necessarily also reflected in the main contributing orbitals of these three molecules in S_1_ and S_2_. To better investigate the electronic transition patterns caused by the change of molecular spatial structure, the main contributing orbitals of the excited states of the three structures are analyzed next to corroborate the above conclusions. In Figure 3, the electron cloud distribution of the primary orbitals that contribute to the S_1_ and S_2_ excited states for the three structures are displayed (with the remaining orbitals depicted in Figure S2). Additionally, Table 1 presents the percentage of the primary contributing orbitals of the S_1_ and S_2_ excited states (>1%) for the three structures. As shown in Figure 3a, the contribution of HOMO to LUMO+1 in the S_1_ excited state of the M, M-1 structure is 81.4%, and the contribution of HOMO to LUMO in the S_2_ excited state is 63.9%. Due to the symmetry of M, M-1 structure, the distribution of LUMO+1 electron cloud is also relatively symmetrical. Compared with HOMO, the position of the electron cloud distribution of LUMO and LUMO+1 has changed. The positions of the electron clouds for LUMO+1 and LUMO are complementary to HOMO. The colors of the upper-left and lower-right regions in LUMO+1 and the lower half of LUMO are opposite to those of HOMO. This suggests that the S_0_→S_1_ and S_0_→S_2_ transition processes are charge-transfer excitations with distinct transition characteristics. In Figure 3b, the contribution of HOMO to LUMO in the S_1_ excited state of the M, M-4 structure is 95.2% and the contribution of HOMO to LUMO+1 in the S_2_ excited state is 62.0%. After introducing the nitrogen heterocycle at the bay position, the symmetry of the molecule was broken, resulting in a significant change in the electron cloud distribution of LUMO and LUMO+1 compared to HOMO. As shown in Figure 3b, the electron clouds of HOMO are distributed on the left and right sides of the structure, while the electron clouds of LUMO are distributed on the right segment of the structure and the electron clouds of LUMO+1 are distributed on the left segment of the structure. During the charge transfer excitation from S_0_→S_1_ and S_0_→S_2_, the distribution of electron clouds is deflected, indicating a disruption in the structure’s symmetry. In Figure 3c, the contribution from HOMO to LUMO+1 in S_1_ excited state of M, M-5 structure is 98.5%, and the contribution from HOMO to LUMO+1 in S_2_ excited state is 59.9%. After introducing two nitrogen heterocycles at the bay position, the structure becomes symmetric, and electron cloud distribution is roughly left-right symmetric. The LUMO+1 electron cloud appears at a position complementary to HOMO, and the distribution colors of electron clouds in both the upper-left and lower-right regions of LUMO+1 are roughly opposite to those of HOMO, with obvious transition characteristics, indicating that both S_0_→S_1_ and S_0_→S_2_ transition processes are dominated by charge transfer excitation. The charge-transfer excitations of the three structures can be explained by analyzing and comparing the orbital patterns of the S_1_ and S_2_ excited states. Through this analysis, we can understand how the electron cloud distribution plays a role in these excitations. The introduction of nitrogen heterocycles disrupts the structural symmetry, leading to differences in the electronic excitation behavior. This finding is consistent with the results discussed previously.

**Figure 4:**
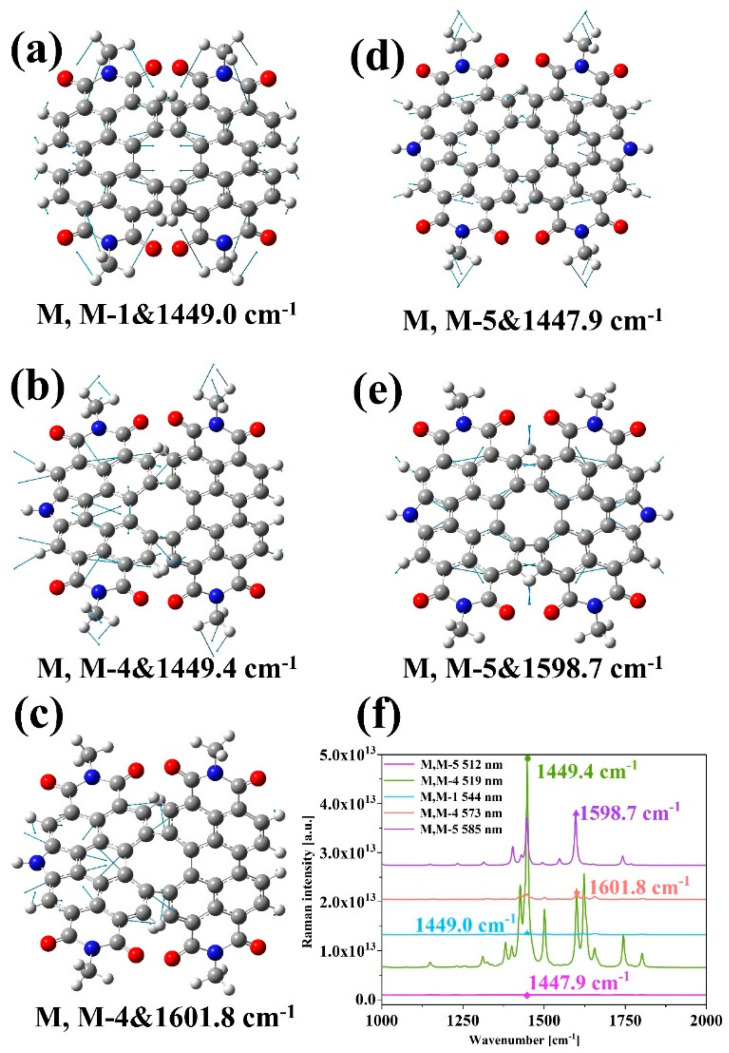
Pre-resonance Raman spectrum (**f**) and vibrational mode diagrams (**a**–**e**) of the three structures. The gray atoms are C, the red atoms are O, the blue atoms are N, and the white atoms are H.

The resonance Raman spectra of three structures were calculated for the S_1_ and S_2_ excited state positions, as shown in Figure 4f. Additionally, Figure S3 displays the static Raman spectrum and vibrational mode diagrams for the three structures. From Figure 4f, it can be seen that although the excitation of the structures at all five wavelengths has the effect of enhancing the Raman peaks, the intensity of the Raman spectrum is much higher when the structures M, M-1 at 544 nm, M, M-4 at 573 nm and M, M-5 at 512 nm are excited than in the other cases. The relative intensities of the Raman peaks in the spectrum change because the activity of different vibrational modes is different for the excitation light. For the excitation of M, M-4 at 519 nm, the peak occurs at 1449.4 cm^−1^. The excitation of M, M-5 at 585 nm with the peak occurring at 1598.7 cm^−1^. In Figure 4a–d, the vibrational modes present at the highest Raman peaks are depicted. The hydrogen atoms on the four methyl groups at the structure’s boundary undergo intense vibrations, along with the carbon atoms at the structure’s center, as shown in Figure 4a for M, M-1 at 1449.0 cm^−1^. M, M-4 is at 1449.4 cm^−1^ and 1601.8 cm^−1^; the symmetry of the structure is broken due to the introduction of the nitrogen heterocycles and the vibrations of the atoms are deflected, and most of the atoms vibrating at this time are distributed on the left side of the structure (Figure 4b,c). In contrast, at 1657.4 cm^−1^, the vibrating atoms are mostly distributed on the right side of the structure (Figure S3c). After introducing two nitrogen heterocycles, the vibrations of M, M-5 recover their symmetry at 1447.9 cm^−1^ and 1598.7 cm^−1^ and are no longer deflected towards a specific segment. By calculating the resonance Raman spectrum at the S_1_ and S_2_ excited state positions, their vibrational modes are discussed, indicating that changes in the molecular spatial structure can have an effect on the Raman spectrum.

**Figure 5:**
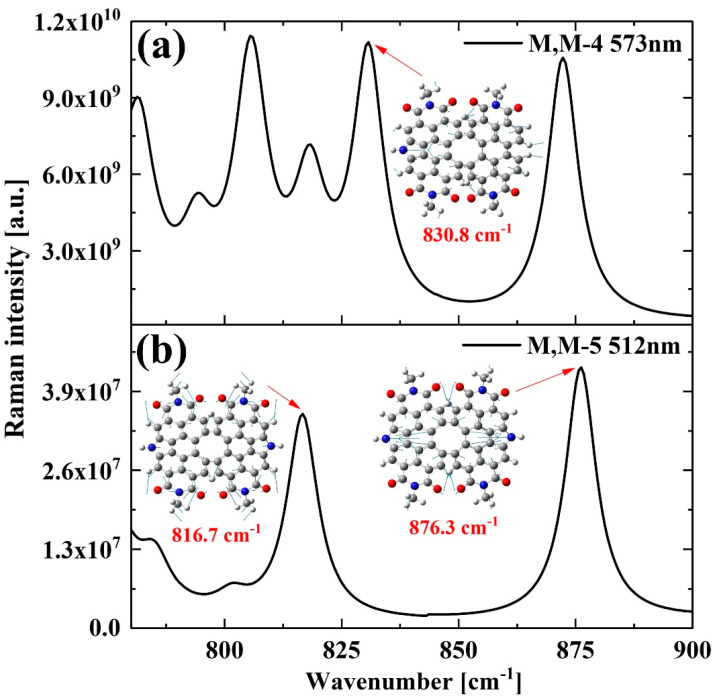
Pre-resonance Raman spectrum and vibrational modes of M, M-4 (**a**) and M, M-5 (**b**) excited at wavelengths of 573 nm and 512 nm, respectively. The gray atoms are C, the red atoms are O, the blue atoms are N, and the white atoms are H.

When excited at 573 nm and 512 nm, M, M-4 and M, M-5 exhibit their vibrational modes and pre-resonance Raman spectrum, as depicted in Figure 5. Due to the doping of nitrogen atoms, novel vibrational modes appear in the M, M-4 and M, M-5 structures: The vibrational modes of the M, M-4 structure at 830.8 cm^−1^ and the M, M-5 structure at 816.7 cm^−1^ and 876.3 cm^−1^ show that the doped nitrogen atoms are vibrating violently, with the direction of vibration pointing toward the central of the structure. This indicates that the doping of nitrogen atoms can affect the vibrational modes of molecules. Combined with the previous section, it can be shown that the changes in molecular spatial structure can affect the Raman spectra.

Figure 1e shows the ECD spectrum of the three structures. Combined with Figure S1d–f, it can be seen that the chiral behavior of the two peaks, M, M-4 and M, M-5, is consistent at around 600 nm. The position of the peak of M, M-1 in the long wavelength region is different, which is related to the shift of the UV–vis spectrum. All three structures have peaks between 500 and 600 nm, and all three peaks are negative at around 470 nm. It is noticeable that the M, M-1 without the addition of nitrogen heterocycles does not show a wave peak before 450 nm (Figure 1e); the M, M-4 structure with one nitrogen heterocycle shows a very weak peak, in which the main contribution is the S_6_ excited state (Figure S1e); the M, M-5 structure with two nitrogen heterocycles shows a strong wave peak, in which the main contribution is the S_5_ excited state (Figure S1d). It is found that, structurally, the appearance of new wave peaks in the short-wave region is caused by the addition of nitrogen atoms, thus exhibiting a novel chiral behavior. After the above analysis, it is found that the electron excitation is related to the number of nitrogen atoms.

**Figure 6:**
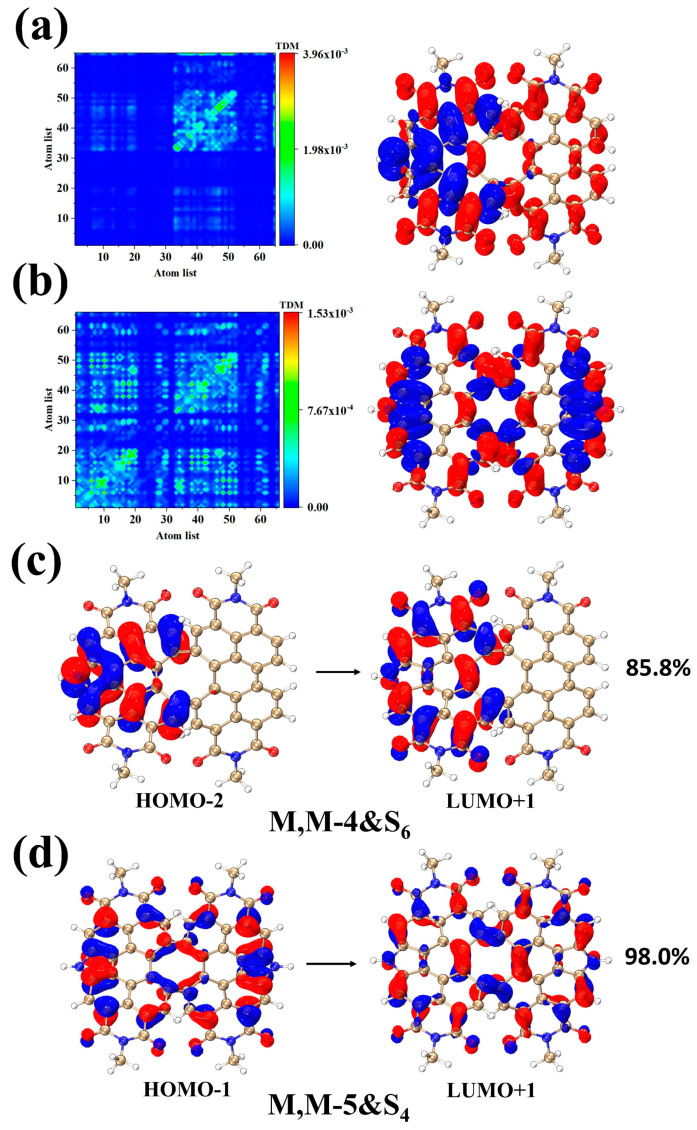
TDM and CDD plots (**a**,**b**) of M, M-4&S_6_ and M, M-5&S_4_. The orbital diagrams of M, M-4&S_6_ and M, M-5&S_4_ (**c**,**d**). The red (blue) isosurfaces represent electron (hole) density, respectively.

This section will focus on the chiral aspects of the M, M-4, and M, M-5 ECD spectra, specifically regarding the significant role of the two excited states S_6_ and S_4_. From the CDD plot of Figure 6a, it can be seen that M, M-4&S_6_ shows a very obvious charge transfer. Because the symmetry of the structure is broken by the introduction of pyrrole at this time, the electron density is mainly distributed on the left fragment containing pyrrole, while the distribution of holes on the right fragment is also significantly less. It can be seen from Figure 6c that the electron clouds of HOMO−2 and LUMO+1 are also mainly concentrated in the left fragment, which is corroborated with the electron-hole density plots. While M, M-5&S_4_ introduce two nitrogen heterocycles, the electron-hole diagram of HOMO−1 orbitals appears novel. It can be seen that the electron-hole distribution direction at the pyrrole one is pointing out of the paper and the other is pointing in the paper. Even though the M, M-5 structure appears symmetrical at the center, the hole density distribution in the paper direction is asymmetrical due to the pyrrole distribution being in the opposite direction. This asymmetry will cause increasing breakage and more severe deflections over time.

**Figure 7:**
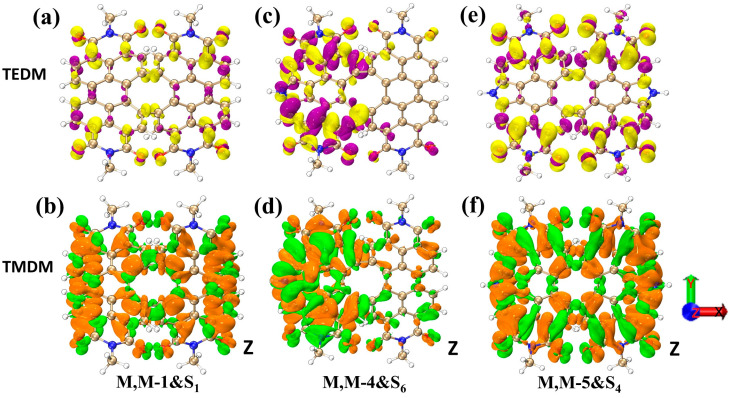
TEDM/TMDM plots of M, M-1&S_1_ (**a**,**b**), M, M-4&S_6_ (**c**,**d**) and M, M-5&S_4_ (**e**,**f**) in the z-direction. The purple (yellow) represents the positive (negative) transition electric dipole moments, respectively, and the green (orange) represents the positive (negative) transition magnetic dipole moments, respectively.

In the following analysis, we will explore the reasons behind the chirality of the M, M-4, and M, M-5 structures by examining the density of TEDMs and TMDMs in various orientations. Referring to Figure 7a,b and Figure S4, it becomes apparent that the distribution of TEDM and TMDM in the M, M-1 structure is uniform in all directions without displaying any anisotropy. For the structure M, M-4, as shown in Figure 7c,d, due to the imbalance in the distribution of the nitrogen heterocycles breaking the symmetry of the structure, the transition electric dipole moments of the left and right sides are deflected, thus causing chirality. As for the structure M, M-5, although the distribution of the nitrogen heterocycles is symmetric, the distribution of the electric dipole moments on the axis of the two newly added nitrogen atoms is actually asymmetric, with the positive transition electric dipole moments closer to the pyrrole and the negative transition electric dipole moments further away from the pyrrole. Although chirality may appear from scratch and from weak to strong, their mechanisms are different. The TEDM/TMDMs of other excited states in all directions are shown in Figures S5–S16.

**Figure 8:**
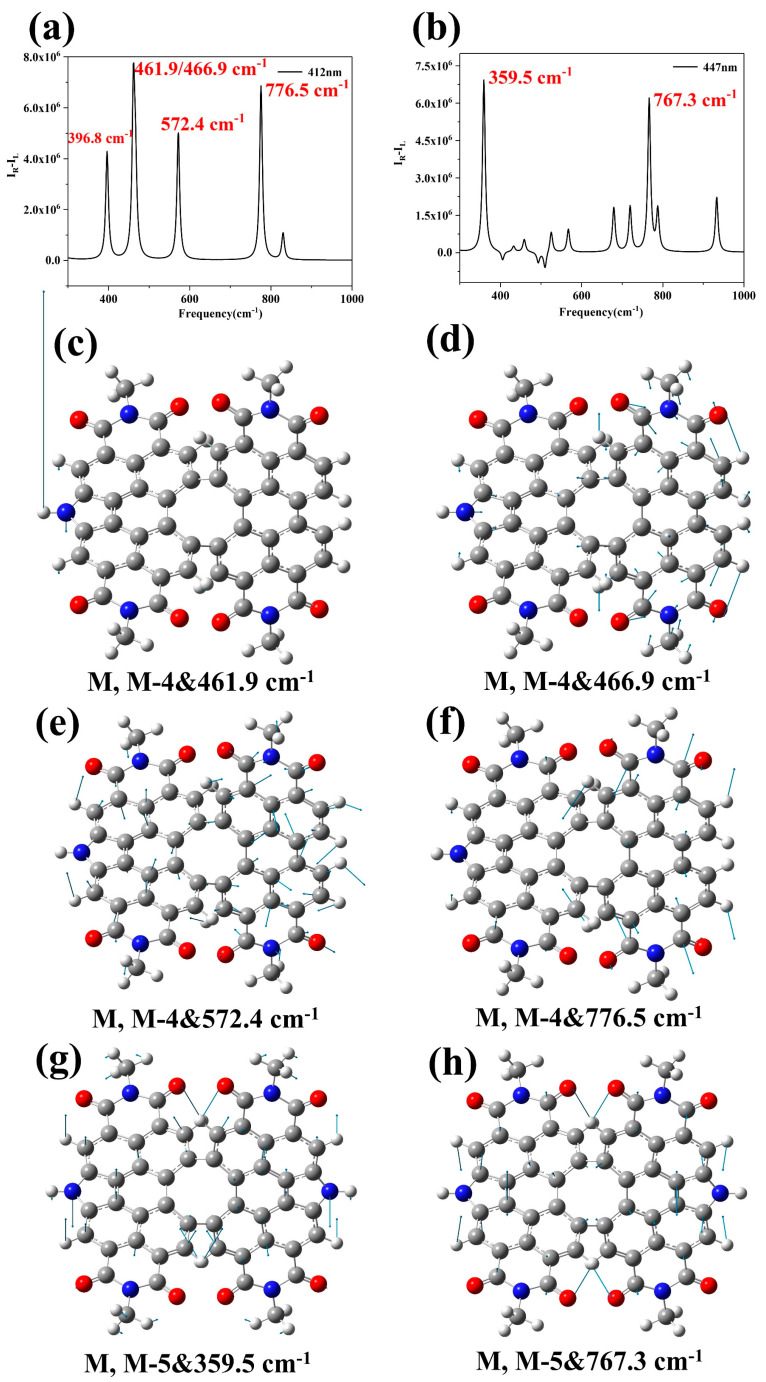
Raman optical activity spectrum and vibrational modes (**c**–**h**) of M, M-4 (**a**) and M, M-5 (**b**). The gray atoms are C, the red atoms are O, the blue atoms are N, and the white atoms are H.

It can be seen that the distribution of TEDM is modified by the symmetry of the nitrogen atoms from the previous discussion. This is because the polarization rate and dipole moment have a direct correlation, and any alteration in the dipole moment will consequently affect Raman optical activity since it relies on the polarization rate. Next, the ROA of M, M-4 and M, M-5 are calculated (Figure 8a,b), and the vibrational modes are given. Visualizing the connection between molecular chirality and ROA spectrum is possible. By observing the vibrational modes, it is apparent that the asymmetry of the nitrogen atom alters the TEDM, leading to a change in the ROA spectrum. For the M, M-4 structure, the four peaks at 396.8 cm^−1^, 466.9 cm^−1^, 572.4 cm^−1^, and 776.5 cm^−1^ are relatively high. The peak at 461.9 cm^−1^ showed the highest activity and the hydrogen atom attached to the nitrogen atom exhibited an extremely strong vibrational intensity (Figure 8c). The distribution of all four vibrational modes is left-right asymmetric due to the variation of the electric dipole moment (Figure 8c–f). For the M, M-5 structure, the two peaks at 359.5 cm^−1^ and 767.3 cm^−1^ are the highest, and they also show activity in the ROA. At 359.5 cm^−1^, the four hydrogen atoms adjacent to the pyrrole vibrate in the upward direction (Figure 8g), but the four hydrogen atoms adjacent to the pyrrole at 767.3 cm^−1^ vibrate in the opposite direction (Figure 8h). As the TEDM is symmetric in the upward and downward direction, the change in TEDM causes the vibrational mode to become active.

## 3. Calculation Methods

In this work, the geometric optimization of the molecular structure was implemented by the Gaussian 16 program [29], based on density functional theory (DFT) [30], B3LYP functional [31], and the 6–31 g(d) basis set [32] combined with DFT-D3 dispersion correction [33]. Using the optimized structure, excited states and UV–vis spectrum calculations were performed using CAM-B3LYP functional [34] and 6–31 g(d) basis set. The frequency analysis was performed at the same calculation level as the optimization. The keyword IOp (9/40 = 4) is used to improve the accuracy of the configuration coefficient output by Gaussian 16. The three structures used in this article do not have virtual frequencies. All wave function analyses including TEDMs/TMDMs, CDD, and TDM were done by Multiwfn program [35]. All 3D plots in this work were plotted by the VMD program [36].

To investigate the electron excitation characteristics, the electronic transition features of three structures are analyzed using hole–electron and TDM methods. The hole–electron analysis is a highly effective and practical method of describing the process of electron transition. It can visually investigate the characteristics of electron excitation in a graphical way. The hole and electron density are defined as:

(1) $$\rho^{\mathrm{hole}}(r)=\rho_{\left(\mathrm{loc}\right)}^{\mathrm{hole}}(r)+\rho_{\left(\mathrm{cross}\right)}^{\mathrm{hole}}(r)$$

(2) $$\rho_{\left(\mathrm{loc}\right)}^{\mathrm{hole}}(r)=\sum_{i\to a}(w_{i}^{a}{)}^{2}\varphi_{i}\varphi_{i}-\sum_{i\leftarrow a}(w{'}_{i}^{a}{)}^{2}\varphi_{i}\varphi_{i}$$

(3) $$\rho_{\left(\mathrm{cross}\right)}^{\mathrm{hole}}(r)=\sum_{i\to a}\sum_{j\neq i\to a}w_{i}^{a}w_{j}^{a}\varphi_{i}\varphi_{j}-\sum_{i\leftarrow a}\sum_{j\neq i\leftarrow a}w{'}_{i}^{a}w{'}_{j}^{a}\varphi_{i}\varphi_{j}$$

(4) $$\rho^{\mathrm{ele}}(r)=\rho_{\left(\mathrm{loc}\right)}^{\mathrm{ele}}(r)+\rho_{\left(\mathrm{cross}\right)}^{\mathrm{ele}}(r)$$

(5) $$\rho_{\left(\mathrm{loc}\right)}^{\mathrm{ele}}(r)=\sum_{i\to a}(w_{i}^{a}{)}^{2}\varphi_{a}\varphi_{a}-\sum_{i\leftarrow a}(w{'}_{i}^{a}{)}^{2}\varphi_{a}\varphi_{a}$$

(6) $$\rho_{\left(\mathrm{cross}\right)}^{\mathrm{ele}}(r)=\sum_{i\to a}\sum_{i\to b\neq a}w_{i}^{a}w_{i}^{b}\varphi_{a}\varphi_{b}-\sum_{i\leftarrow a}\sum_{i\leftarrow b\neq a}w{'}_{i}^{a}w{'}_{i}^{b}\varphi_{a}\varphi_{b}$$

where r is the coordinate vector, φ is the orbital wave function, i or j is the occupied orbital marker, a or b is the empty orbital marker. $\sum {}_{i\to a}$ represents cycling each excited group state, while $\sum {}_{i\leftarrow a}$ represents cycling each de-excited group state. Both the hole distribution $\rho^{\mathrm{hole}}$ and the electron distribution $\rho^{\mathrm{ele}}$ are divided into two parts: the local term and the cross term. The TDM diagram is a matrix containing characteristic information of electron transitions. The TDM diagram can clearly show the position and quantity of electron transfer during the electron excitation process. It helps understand the intrinsic characteristics of electron excitation.

## 4. Conclusions

In this work, by plotting the UV–vis spectrum, it was found that the introduction of nitrogen heterocycles at the bay position leads to a splitting of the excited state, which results in the appearance of new absorption peaks. The main contributing orbitals of the S_1_ and S_2_ excited states of the three structures were discussed, and the nature of the charge-transfer excitations was explained from the perspective of the electron cloud distributions, and the differences in the electronic excitation behaviors due to the introduction of nitrogen heterocycles to break the structural symmetry were clarified. The vibrational modes of S_1_ and S_2_ were discussed by calculating the pre-resonance Raman spectra at the positions of their excited states, thus demonstrating that changes in the spatial structure can affect the Raman spectra. The study of ECD spectra reveals that the short-wave region exhibits a new chiral behavior with new wave peaks due to the incorporation of nitrogen heterocycles and the number of nitrogen heterocycles. By analyzing the densities of TEDMs/TMDMs in different orientations, it can be seen that the change of chirality goes through two processes, from absent to present and from weak to strong, but their mechanisms are completely different: the M, M-4 structure produces chirality due to the deflection of the TEDM on the left and right sides, whereas the M, M-5 structure produces chirality because of the asymmetric distribution of the TEDM in the axes of the two newly added nitrogen atoms. Finally, the ROA spectra of M, M-4 and M, M-5 show an effect on Raman optical activity due to changes in the dipole moment. This study will have a significant impact on the application of carbon-based nanomaterials in organic electronics, solar cells, and optoelectronics.

## Figures and Tables

**Table 1:** Percentage of major contributing orbitals in S_1_, S_2_ excited states in the three structures (>1%).

	Excited States	MOs	Contribution Ratio
M, M-1	S_1_	HOMO→LUMO+1	81.4%
		HOMO−1→LUMO	17.5%
	S_2_	HOMO→LUMO	69.3%
		HOMO−1→LUMO+1	29.6%
M, M-4	S_1_	HOMO→LUMO	95.2%
		HOMO→LUMO+1	1.88%
		HOMO−1→LUMO	1.67%
	S_2_	HOMO→LUMO+1	62.0%
		HOMO−1→LUMO	31.3%
		HOMO−1→LUMO+1	5.2%
M, M-5	S_1_	HOMO→LUMO+1	98.5%
	S_2_	HOMO→LUMO+1	59.9%
		HOMO−1→LUMO	38.9%

## Data Availability

Not applicable.

## Supplementary Materials

The following supporting information can be downloaded at: https://www.mdpi.com/article/10.3390/molecules28186645/s1, Figure S1. UV-vis (a–c) spectrums and ECD spectrums (d–f) of the three structures; Figure S2. The orbital contribution plots of S_1_, S_2_ excited states of the three structures (>1%); Figure S3. Static Raman spectrum (d) and vibrational mode (a–c) of the three structures; Figure S4. TEDM\TMDM plots of M, M-1&S_1_ in X and Y directions, with purple (yellow) representing positive (negative) transition electric dipole moments and green (orange) representing positive (negative) transition magnetic dipole moments, respectively; Figure S5. TEDM\TMDM plots of M, M-1&S_2_ in X, Y and Z directions, with purple (yellow) representing positive (negative) transition electric dipole moments and green (orange) representing positive (negative) transition magnetic dipole moments, respectively; Figure S6. TEDM\TMDM plots of M, M-1&S_3_ in X, Y and Z directions, with purple (yellow) representing positive (negative) transition electric dipole moments and green (orange) representing positive (negative) transition magnetic dipole moments, respectively; Figure S7. TEDM\TMDM plots of M, M-1&S_4_ in X, Y and Z directions, with purple (yellow) representing positive (negative) transition electric dipole moments and green (orange) representing positive (negative) transition magnetic dipole moments, respectively; Figure S8. TEDM\TMDM plots of M, M-4&S_1_ in X, Y and Z directions, with purple (yellow) representing positive (negative) transition electric dipole moments and green (orange) representing positive (negative) transition magnetic dipole moments, respectively; Figure S9. TEDM\TMDM plots of M, M-4&S_2_ in X, Y and Z directions, with purple (yellow) representing positive (negative) transition electric dipole moments and green (orange) representing positive (negative) transition magnetic dipole moments, respectively; Figure S10. TEDM\TMDM plots of M, M-4&S_3_ in X, Y and Z directions, with purple (yellow) representing positive (negative) transition electric dipole moments and green (orange) representing positive (negative) transition magnetic dipole moments, respectively; Figure S11. TEDM\TMDM plots of M, M-4&S_4_ in X, Y and Z directions, with purple (yellow) representing positive (negative) transition electric dipole moments and green (orange) representing positive (negative) transition magnetic dipole moments, respectively; Figure S12. TEDM\TMDM plots of M, M-4&S_6_ in X and Y directions, with purple (yellow) representing positive (negative) transition electric dipole moments and green (orange) representing positive (negative) transition magnetic dipole moments, respectively; Figure S13. TEDM\TMDM plots of M, M-5&S_1_ in X, Y and Z directions, with purple (yellow) representing positive (negative) transition electric dipole moments and green (orange) representing positive (negative) transition magnetic dipole moments, respectively; Figure S14. TEDM\TMDM plots of M, M-5&S_3_ in X, Y and Z directions, with purple (yellow) representing positive (negative) transition electric dipole moments and green (orange) representing positive (negative) transition magnetic dipole moments, respectively; Figure S15. TEDM\TMDM plots of M, M-5&S_4_ in X and Y directions, with purple (yellow) representing positive (negative) transition electric dipole moments and green (orange) representing positive (negative) transition magnetic dipole moments, respectively; Figure S16. TEDM\TMDM plots of M, M-5&S_5_ in X, Y and Z directions, with purple (yellow) representing positive (negative) transition electric dipole moments and green (orange) representing positive (negative) transition magnetic dipole moments, respectively.
