# Supplementary material for: Study on Asymmetric Vibrational Coherent Magnetic Transitions and Origin of Fluorescence in Symmetric Structures
Source: Molecules. 2023 Sep 15;28(18):6645. doi: 10.3390/molecules28186645 (PMC10534477; doi:10.3390/molecules28186645)
Supplement: Supplementary file 1 [file molecules-28-06645-s001.zip › molecules-2564210-supplementary.pdf]

Study on Asymmetric Vibrational Coherent Magnetic Transitions and Origin of Fluorescence in Symmetric Structures

Lulu Sun †, Ning Li †, Ji Ma * and Jingang Wang *

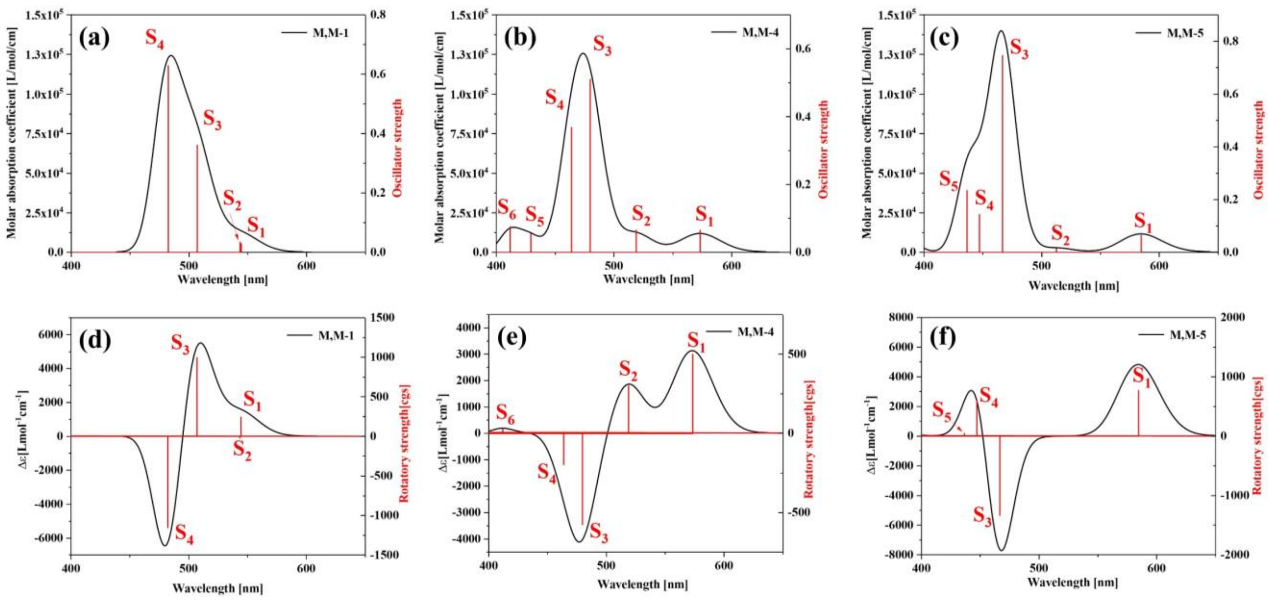

Figure S1. UV-vis (a-c) spectrums and ECD spectrums (d-f) of the three structures.

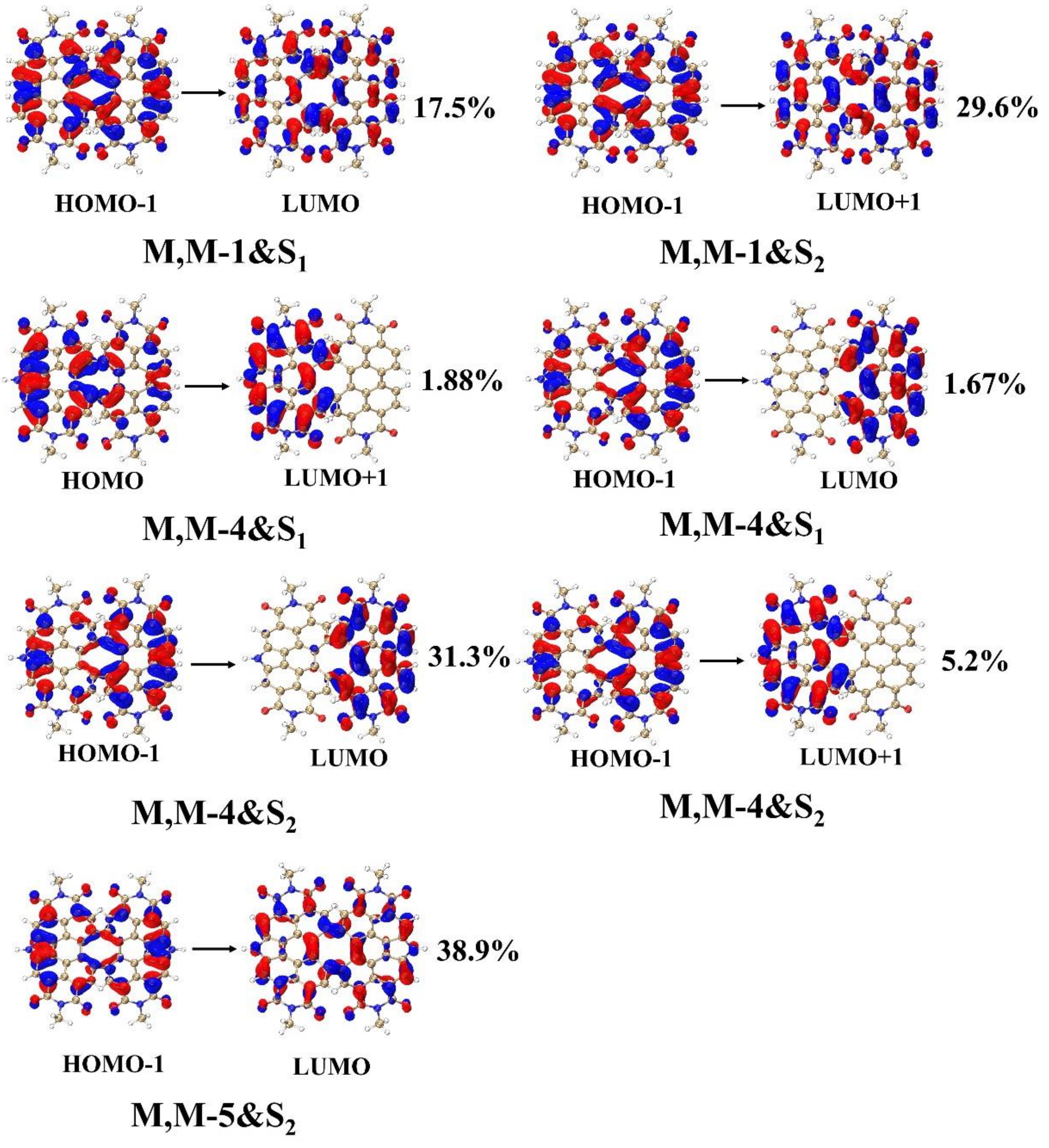

Figure S2. The orbital contribution plots of S_1, S_2 excited states of the three structures (>1%).

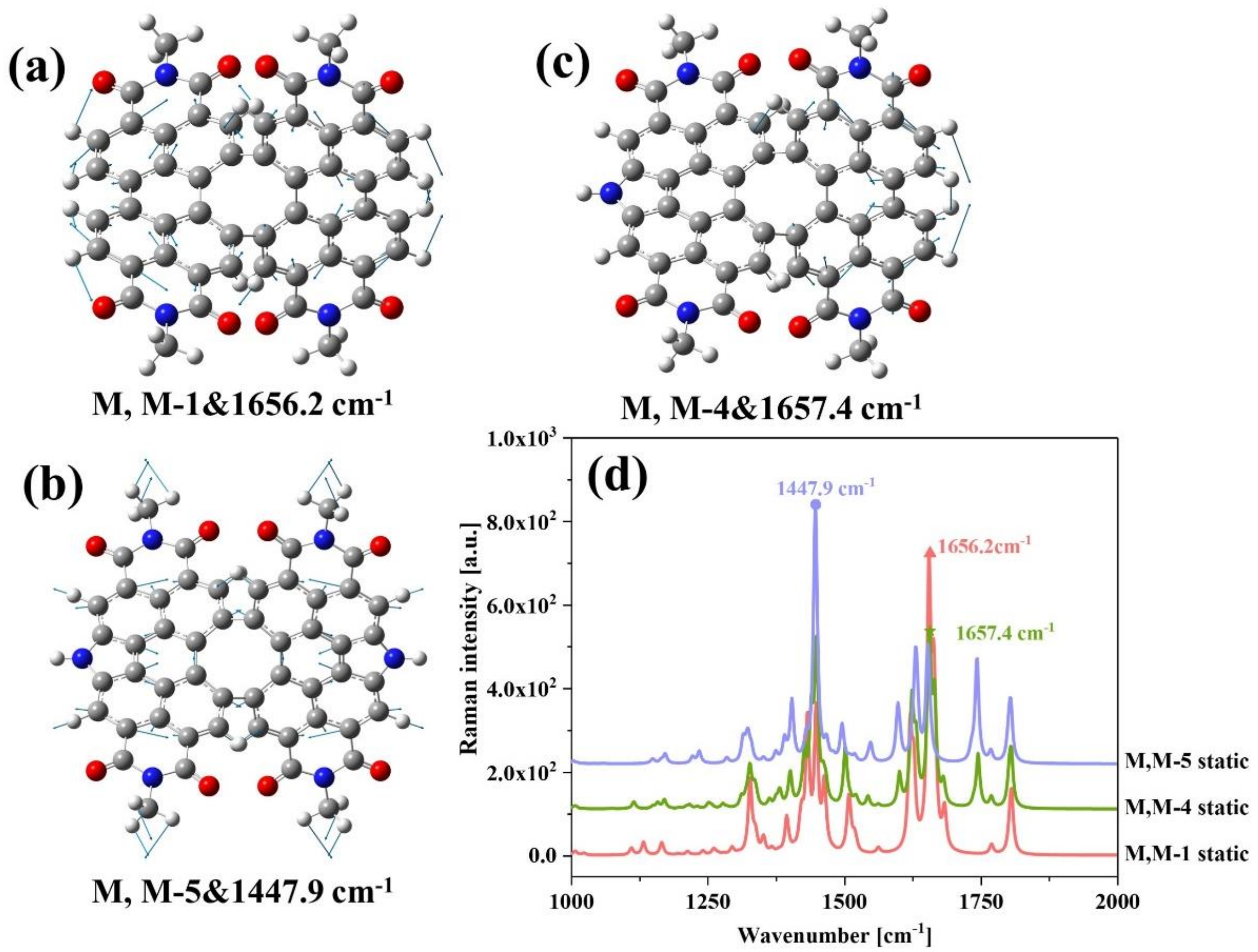

Figure S3. Static Raman spectrum (d) and vibrational mode (a-c) of the three structures.

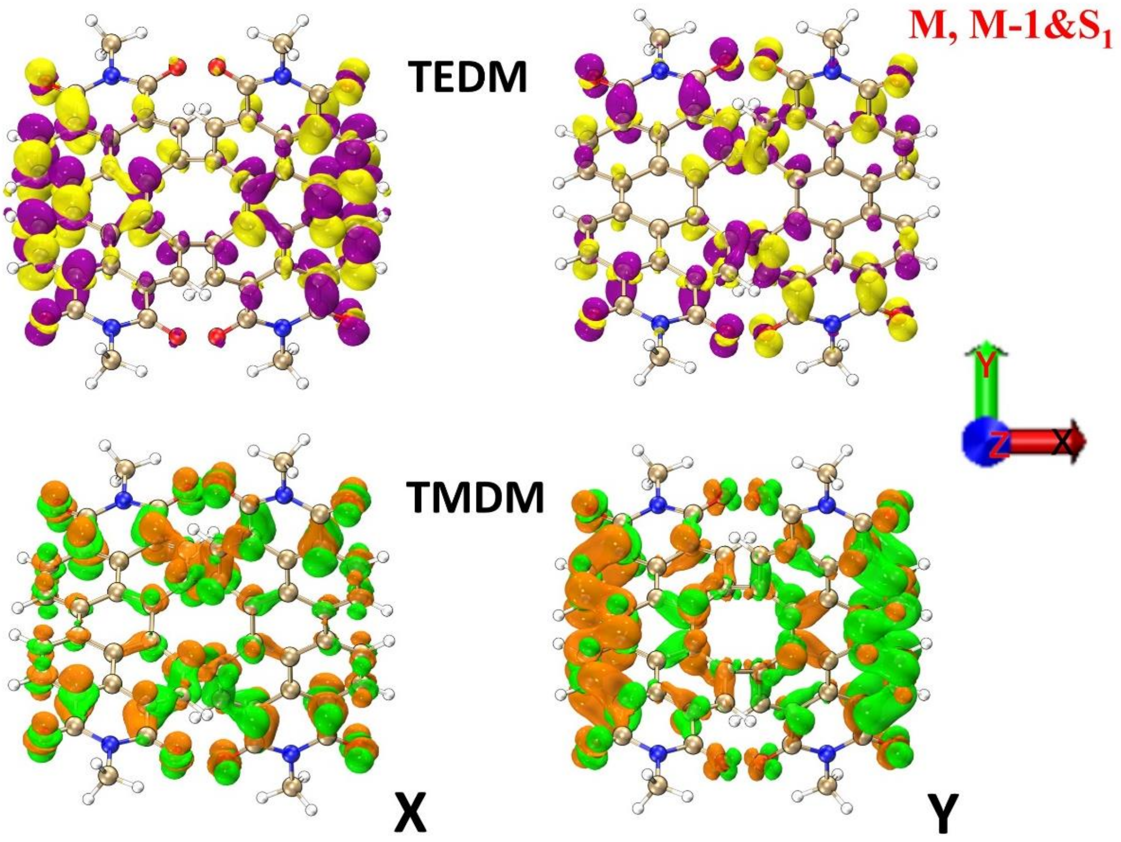

Figure S4. TEDM\TMDM plots of M, M-1&S_1 in X and Y directions, with purple (yellow) representing positive (negative) transition electric dipole moments and green (orange) representing positive (negative) transition magnetic dipole moments, respectively.

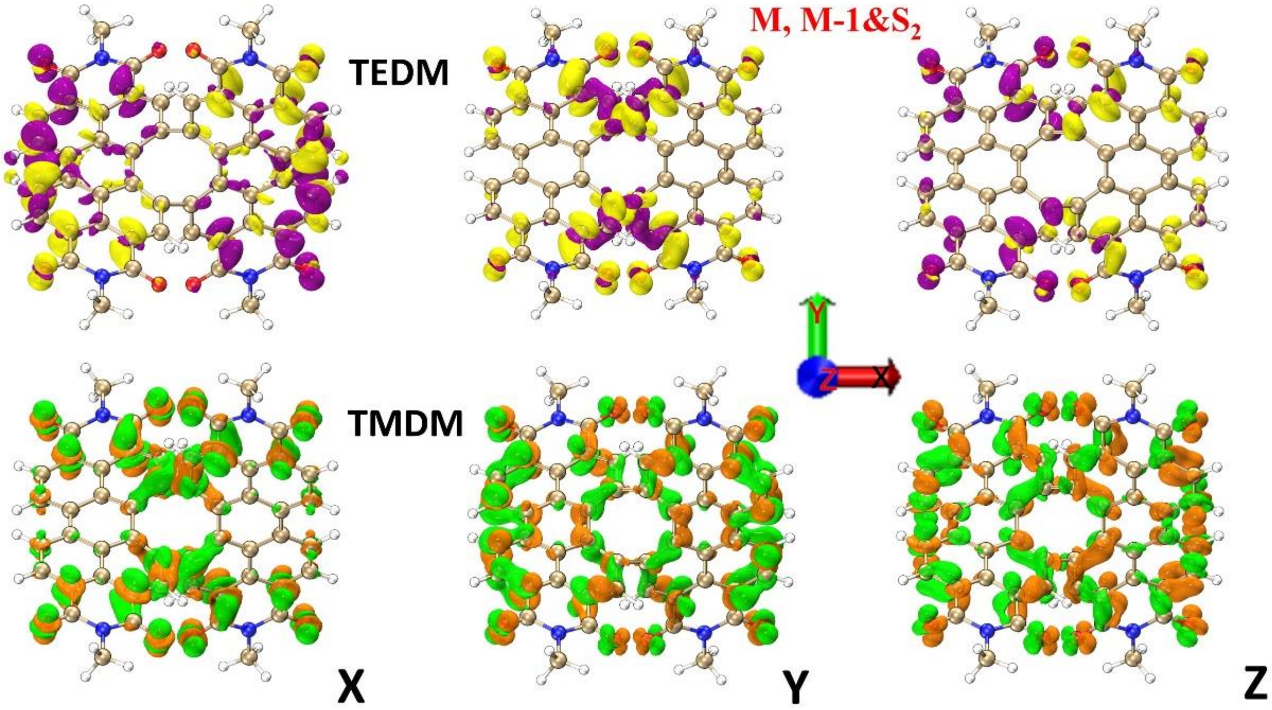

Figure S5. TEDM\TMDM plots of M, M-1&S_2 in X, Y and Z directions, with purple (yellow) representing positive (negative) transition electric dipole moments and green (orange) representing positive (negative) transition magnetic dipole moments, respectively.

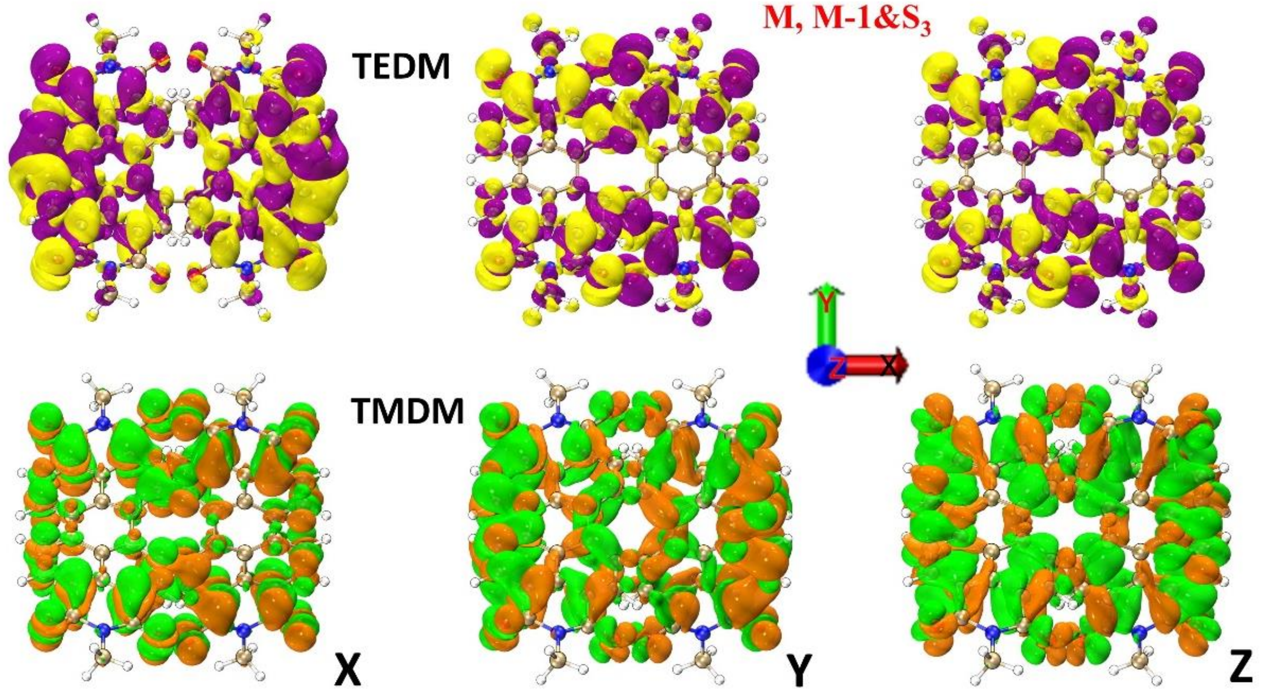

Figure S6. TEDM\TMDM plots of M, M-1&S_3 in X, Y and Z directions, with purple (yellow) representing positive (negative) transition electric dipole moments and green (orange) representing positive (negative) transition magnetic dipole moments, respectively.

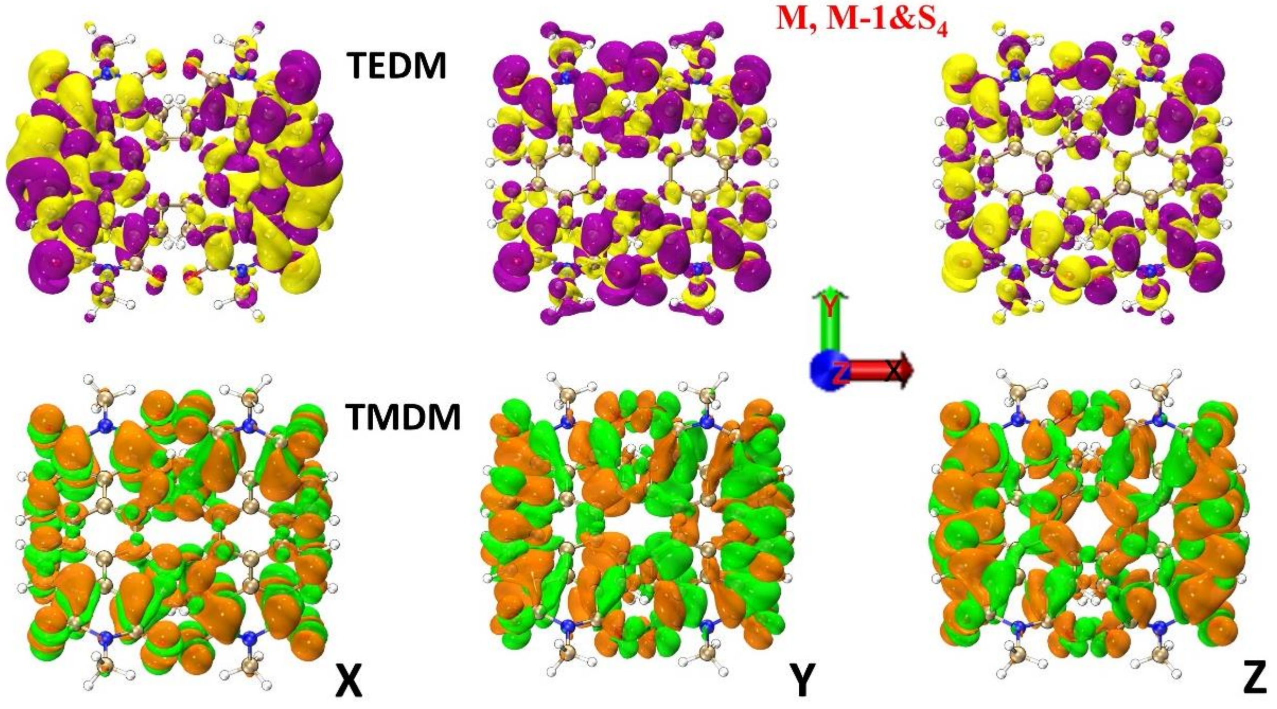

Figure S7. TEDM\TMDM plots of M, M-1&S_4 in X, Y and Z directions, with purple (yellow) representing positive (negative) transition electric dipole moments and green (orange) representing positive (negative) transition magnetic dipole moments, respectively.

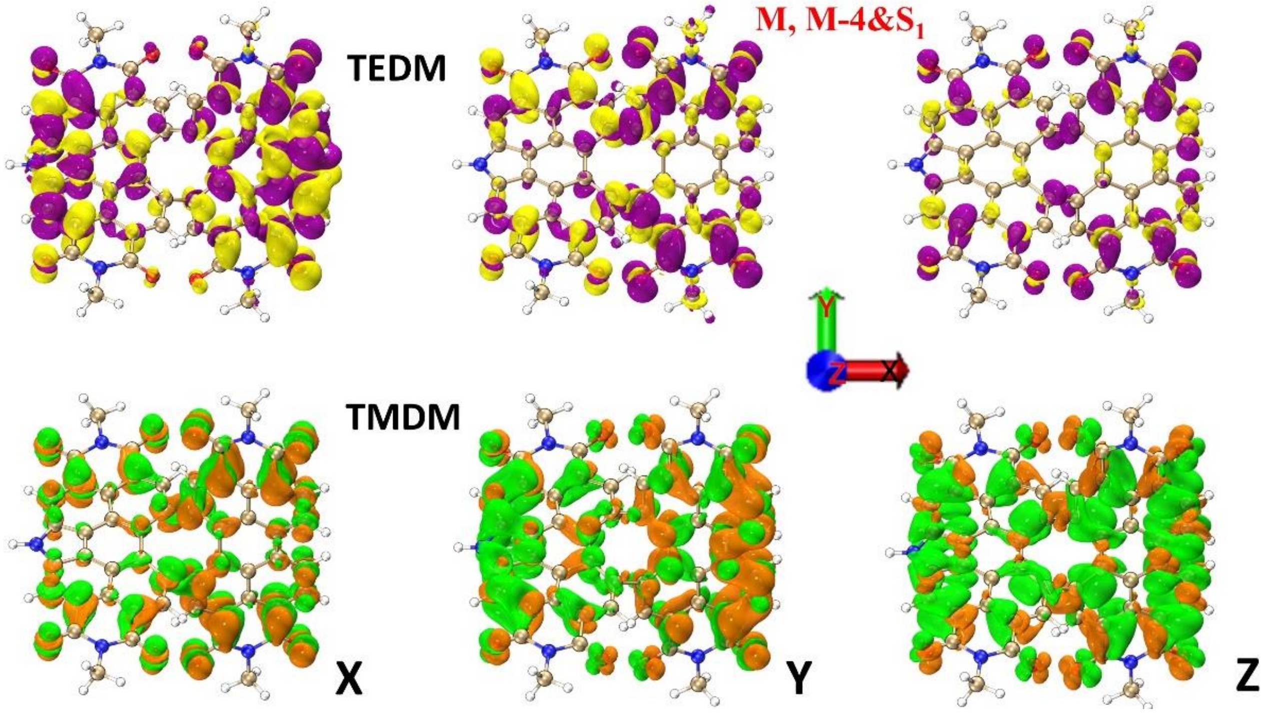

Figure S8. TEDM\TMDM plots of M, M-4&S_1 in X, Y and Z directions, with purple (yellow) representing positive (negative) transition electric dipole moments and green (orange) representing positive (negative) transition magnetic dipole moments, respectively.

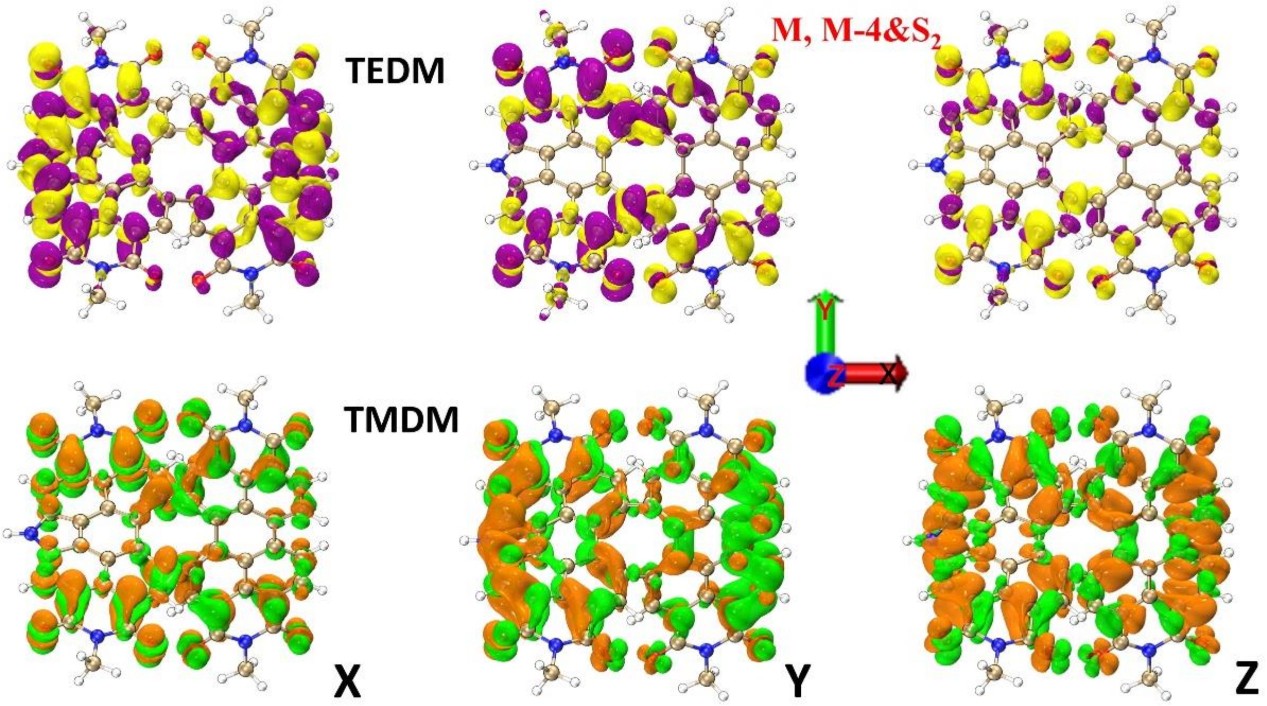

Figure S9. TEDM\TMDM plots of M, M-4&S_2 in X, Y and Z directions, with purple (yellow) representing positive (negative) transition electric dipole moments and green (orange) representing positive (negative) transition magnetic dipole moments, respectively.

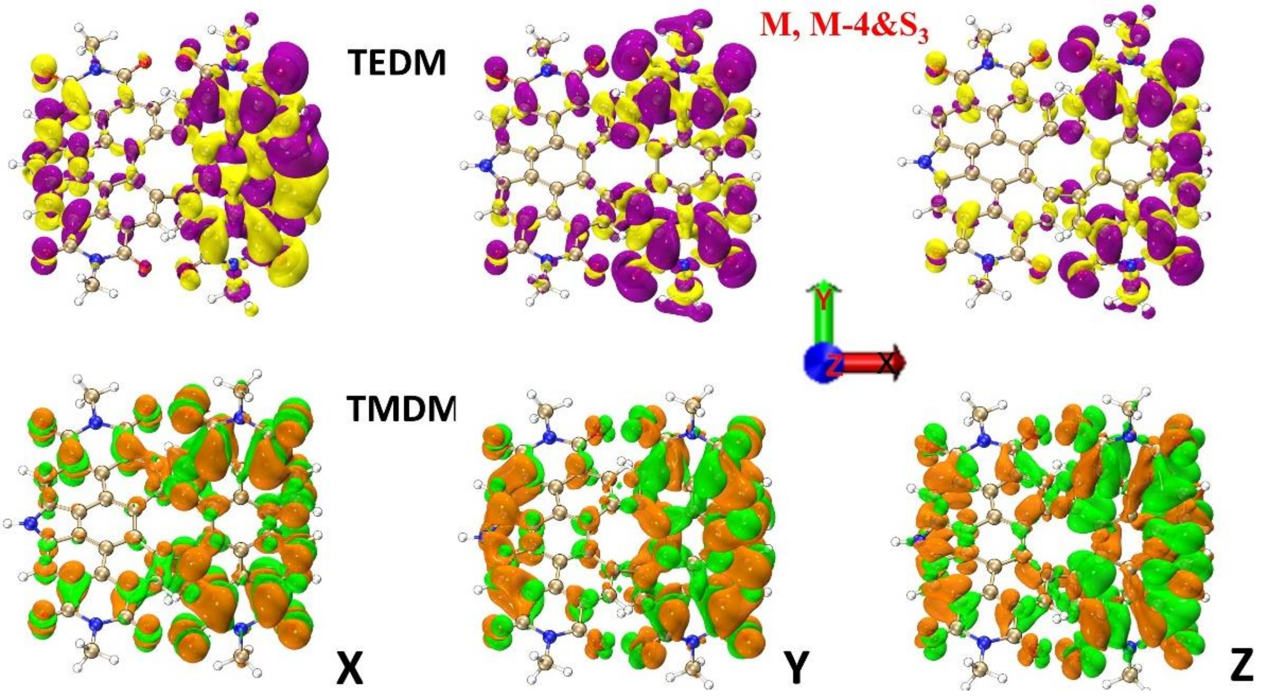

Figure S10. TEDM\TMDM plots of M, M-4&S_3 in X, Y and Z directions, with purple (yellow) representing positive (negative) transition electric dipole moments and green (orange) representing positive (negative) transition magnetic dipole moments, respectively.

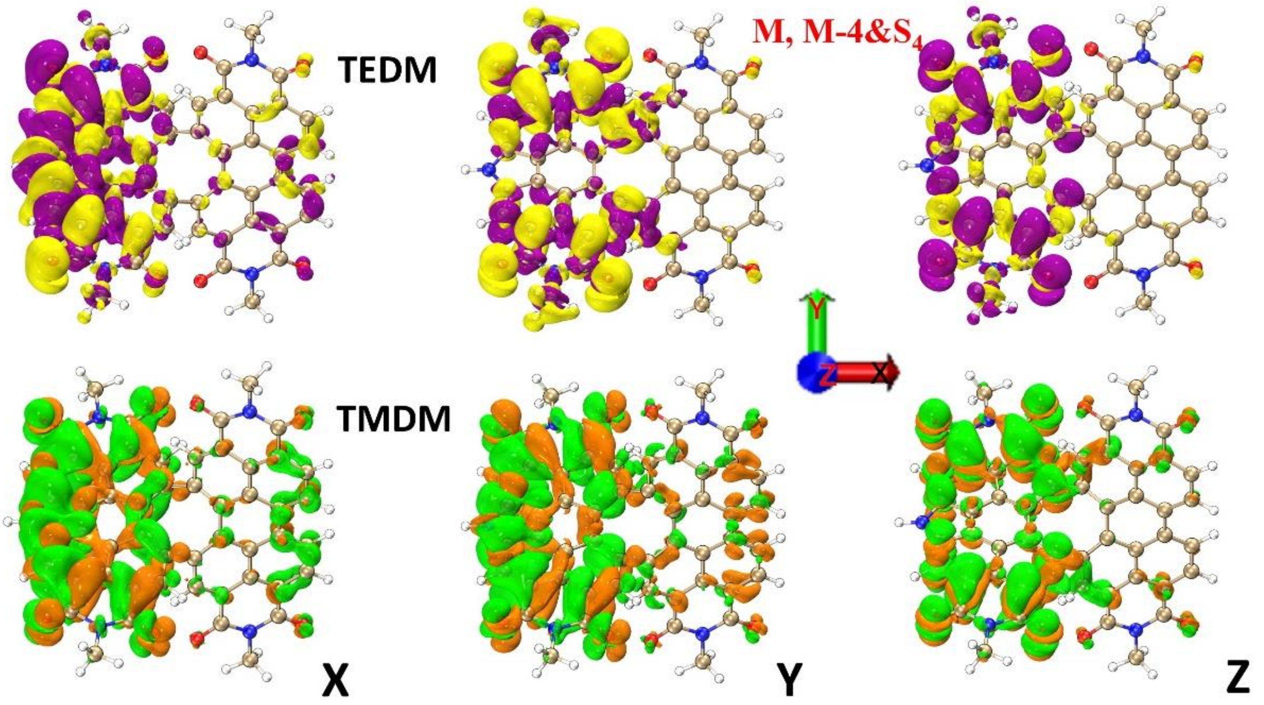

Figure S11. TEDM\TMDM plots of M, M-4&S_4 in X, Y and Z directions, with purple (yellow) representing positive (negative) transition electric dipole moments and green (orange) representing positive (negative) transition magnetic dipole moments, respectively.

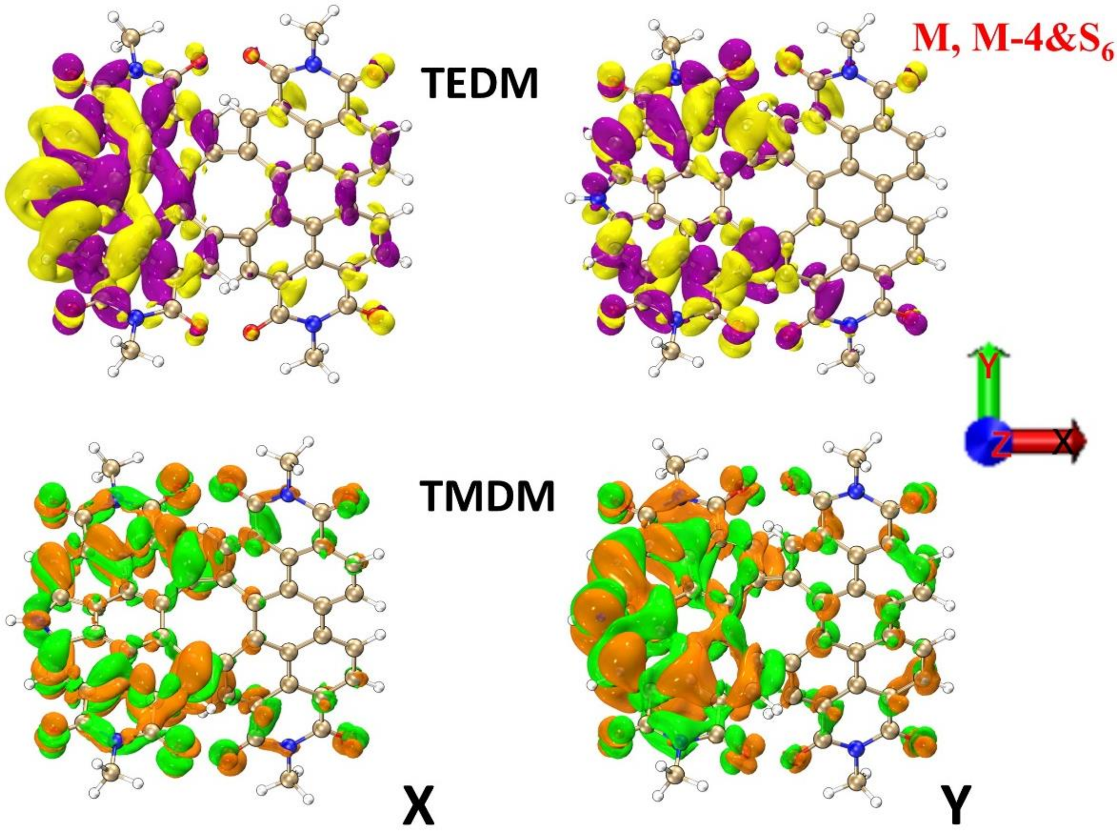

Figure S12. TEDM\TMDM plots of M, M-4&S_6 in X and Y directions, with purple (yellow) representing positive (negative) transition electric dipole moments and green (orange) representing positive (negative) transition magnetic dipole moments, respectively.

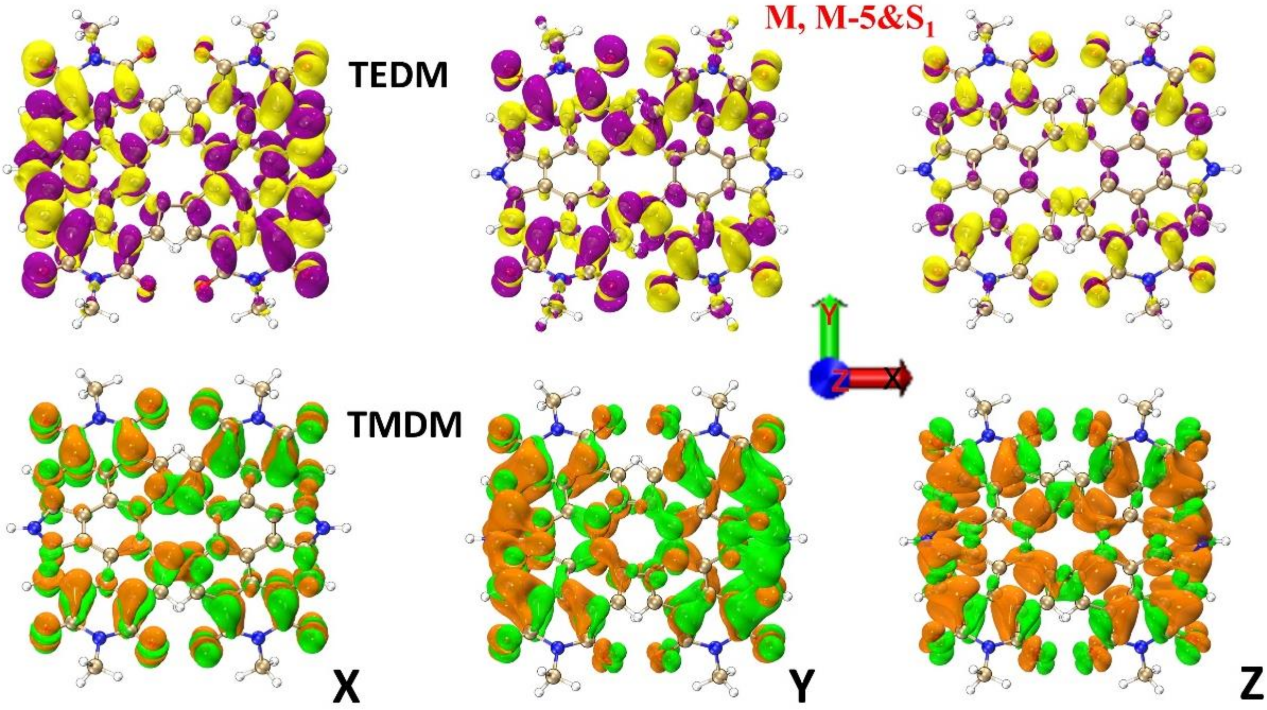

Figure S13. TEDM\TMDM plots of M, M-5&S_1 in X, Y and Z directions, with purple (yellow) representing positive (negative) transition electric dipole moments and green (orange) representing positive (negative) transition magnetic dipole moments, respectively.

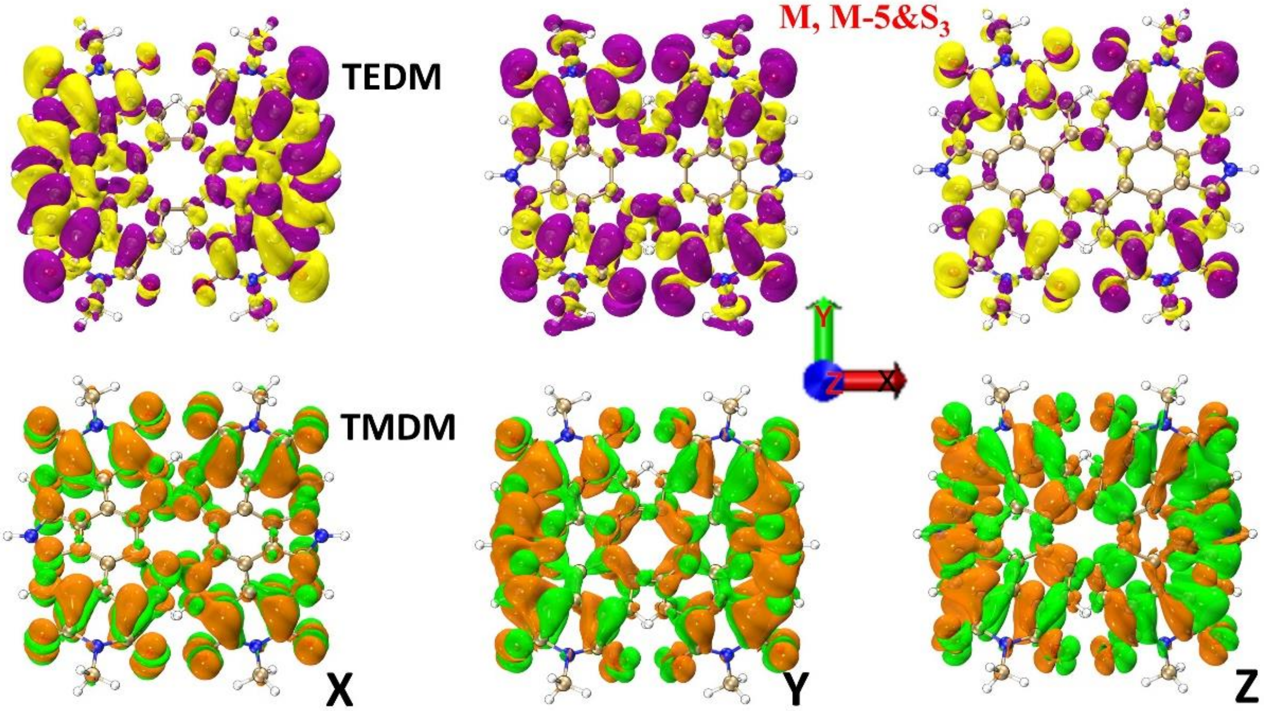

Figure S14. TEDM\TMDM plots of M, M-5&S_3 in X, Y and Z directions, with purple (yellow) representing positive (negative) transition electric dipole moments and green (orange) representing positive (negative) transition magnetic dipole moments, respectively.

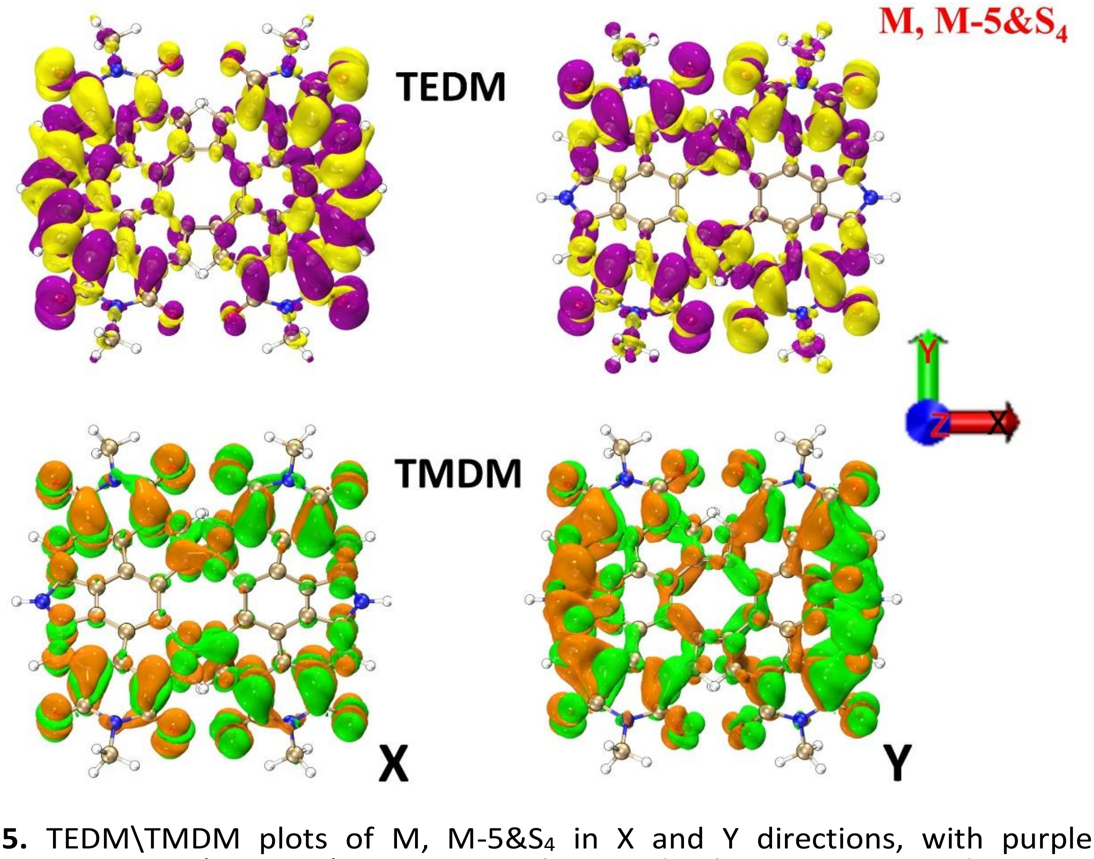

Figure S15. TEDM\TMDM plots of M, M-5&S_4 in X and Y directions, with purple (yellow) representing positive (negative) transition electric dipole moments and green (orange) representing positive (negative) transition magnetic dipole moments, respectively.

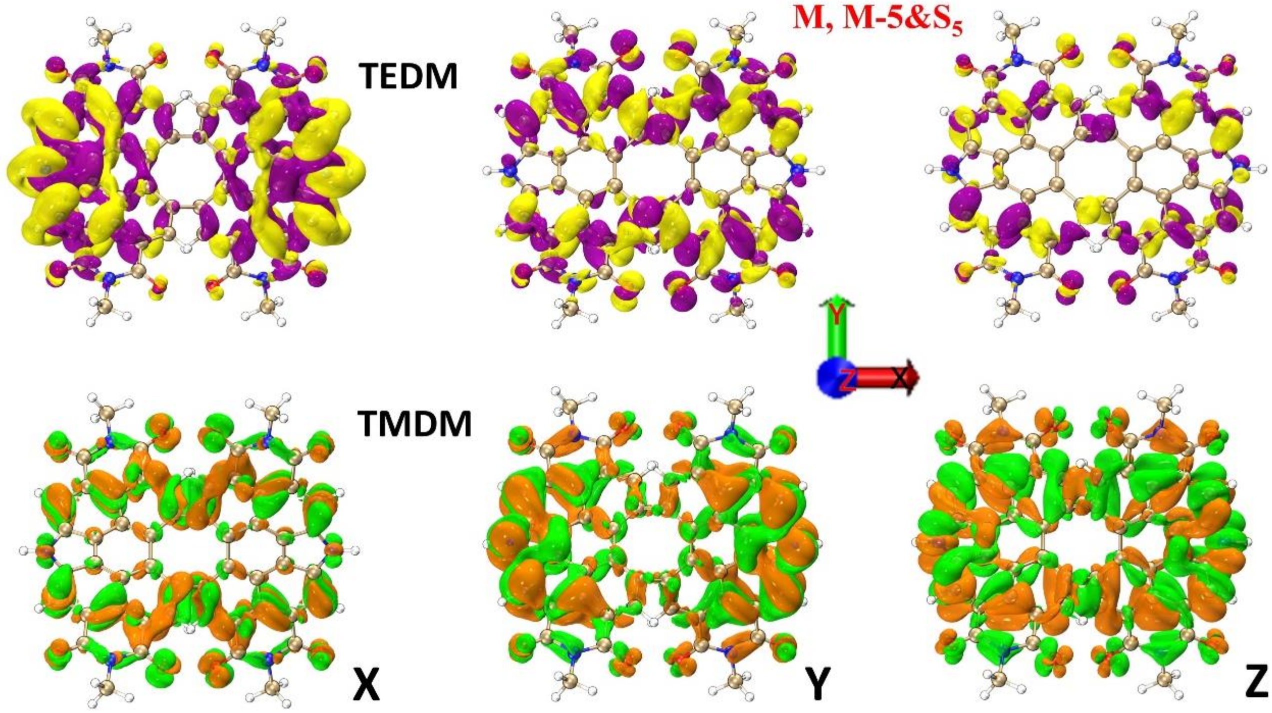

Figure S16. TEDM\TMDM plots of M, M-5&S_5 in X, Y and Z directions, with purple (yellow) representing positive (negative) transition electric dipole moments and green (orange) representing positive (negative) transition magnetic dipole moments, respectively.
